# The Effect of Three Different Root Canal Irrigant Protocols for Removing Smear Layer on the Apical Microleakage of AH26 Sealer

**Published:** 2008-07-10

**Authors:** Ali Reza Farhad, Behnaz Barekatain, Ali Reza Koushki

**Affiliations:** 1 *Department of Endodontics, Torabinejad Research Center, Dental School, Isfahan University of Medical Sciences, Isfahan, Iran*; 2 *General Practitioner, Isfahan, Iran*

**Keywords:** AH26 Sealer, Citric Acid, Dental Leakage, EDTA, Irrigation, Smear Layer

## Abstract

**INTRODUCTION:** The purpose of this *in vitro *study was to assess the apical microleakage of AH26 sealer when three different root canal irrigant regimens were used.

**MATERIALS AND METHODS:** Eighty single-rooted human teeth were randomly divided into three experimental (n=20) and two control groups (n=10). NaOCl was used as irrigant during instrumentation, and apical patency was ensured in all teeth. Final irrigation was implemented as follow: group A- 17% EDTA + 5.25% NaOCl, Group B- 7% citric acid + 5.25% NaOCl, and group C- 20% citric acid + 5.25% NaOCl. The experimental and negative control groups were obturated by laterally condensed gutta-percha with AH26 sealer. The positive control group was obturated without sealer. The teeth were stored in 100% humidity and 37ºC for 48 hours. In the experimental groups and positive control group, the root surfaces except for the apical 2 mm were covered with nail polish and sticky wax. In the negative control group, the roots were completely covered. The samples were then immersed in 2% methylene blue dye for 72 hours at 37ºC. After that the roots were sectioned longitudinally and the dye penetration was measured. The results were statistically analyzed by One-way Variance and Post Hoc Tukey tests.

**RESULTS:** Statistically significant difference was found between groups (P<0.05). Group C showed the least (1.072 mm) and group A showed the most (2.072 mm) amount of dye penetration.

**CONCLUSION:** When a resin-based sealer is used for the obturation of the root canal system, it is better to use a citric acid irrigant instead of EDTA to remove the smear layer and to improve the apical seal.

## Introduction

The ultimate aim of root canal treatment is to prepare a clean, bacteria and debris-free canal for obturation. A three-dimensional seal of root canal system to prevent coronal and apical microleakage of bacteria and their by-products is an important phase in achieving good prognosis for endodontic treatments. Ingle believes most unsuccessful cases of root canal treatment are caused by percolation of fluid from inflamed periapical tissue into improperly obturated canals (1). Kakehashi *et al. *states that microorganisms and their by-products are the main etiological factors of pulpal and periradicular pathosis, and a successful endodontic treatment depends on the elimination of these bacteria with the prevention of future recontamination (2). Allen (3) and Strindberg (4) have shown that incomplete seal of the root canal system is one of the most important causes of long term treatment failures and they propose a fluid-tight seal for the entire root canal system.

A number of studies have shown the formation of a layer of a sludge material (smear layer) over the surfaces of instrumented root canal walls (5-6). Smear layer is an amorphous, irregular entity containing organic (pulp tissue, bacteria) and inorganic (dentin) material. The smear layer is composed of two separate layers: (I) a superficial layer which is loosely attached to the underlying dentin and (II) a deeper layer of debris compacted into the dentinal tubules called the smear plug (6). Mc Comb and Smith (5) suggest that smear layer can create a space between the inner wall of root canal and the obturating materials, thus preventing the complete locking and adherence of the root canal filling materials into the dentinal tubules. Pashley *et al. *believes that smear layer contains bacteria and bacterial by-products and thus must be completely removed from the root canal system (7). Haapasalo *et al. *suggests that removal of the smear layer can allow intracanal medicaments to penetrate the dentinal tubules for better disinfection (8).

According to Torabinejad *et al. *(9) smear layer is one of the factors that can adversely affect the apical and coronal microleakage compromising the long term success of the treatment. These unwanted layers of organic and inorganic materials should be removed before obturation of the root canal system. Many investigations have demonstrated that removal of smear layer causes a better adherence and penetration of sealer into the dentinal tubules preventing apical/coronal microleakage (10-15).

Resin-based sealers not only can penetrate the dentinal tubules, but also can adhere to the exposed dentin surface. A better adherence of resin-based sealers might be obtained if a larger surface area of dentin is created by the irrigation regimen. Different irrigation protocols in removing the smear layer create different dentin surface structures microscopically. Thus, one can assume that a particular surface structure of dentin is more suitable for a specific sealer. The purpose of this study was to assess the apical microleakage of AH26 sealer when three different root canal irrigant regimens (17% EDTA, 7% citric acid, and 20% citric acid) were used to remove the smear layer.

## MATERIALS AND METHODS

This experimental study selected 80 freshly extracted human teeth (maxillary centrals and canines, mandibular premolars). The teeth had the following characteristics: 1) all had single canals, 2) the roots did not have fractures, caries and/or open or resorbed apices and finally 3) all roots were straight or had slight curvature (5-10 degree). In order to clean the surface of the teeth, they were placed in 2.5% NaOCl for 48 hours. All teeth were radiographically investigated to ensure that they have single canals and that there are no calcifications. To standardize the samples, the crowns of the teeth were cut at CEJ using a diamond disk so that an average length of the remaining roots was approximately 13-15 mm. Before instrumentation, the samples were randomly divided into three experimental groups A, B, and C (n=20) and two control groups (n=10). All teeth were kept in normal saline solution during the experiment. A #10 K-file (Mani, Japan) and rubber stop was used to determine the working length for each root.

All roots were instrumented up to a #40 K-file, and the coronal part was flared up to #70 K-file using step back technique. A #10 K-file was used to ensure apical patency between each file. Full strength NaOCl (5.25%) was used as irrigant during instrumentation of all canals. To remove the smear layer, the following three different final irrigation protocols were used (16-17):

***Group A***: 5mL of 17% EDTA (Merck, Darmstadt, Germany) for 1 min + 5mL of 5.25% NaOCl + 5mL of distilled water.

***Group B***: 5mL of 7% citric acid (Carlo Erba, Milan, Italy) for 1 min + 5mL of 5.25% NaOCl + 5mL of distilled water.

***Group C***: 5mL of 20% citric acid (Carlo Erba, Milan, Italy) for 1 min + 5 mL of 5.25% NaOCl + 5mL of distilled water.

Distilled water was used at the end of irrigation procedure to terminate the action and eliminate any precipitates from the irrigants. After completion of the final irrigation phase, the canals in groups A, B, and C were dried with paper points and then obturated laterally using #40 gutta-percha as master cone and #25 gutta- percha as accessory cones (AriaDent, Asia Chemi Teb Co., Tehran, Iran). AH26 (Dentsply, DeTrey, Konstanz, Germany) was used as sealer in this experiment. In the positive control group, the canals were dried and obturated without sealer, and in the negative control group, the canals were dried and obturated with gutta- percha and sealer.

**Figure 1:**
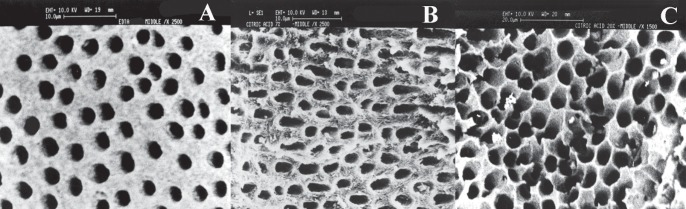
Scanning electrone microscope appearance of dentin surface after irrigation with A) 17% EDTA, B) 7% citric acid, and C) 20% citric acid

**Table 1:** Mean dye leakage (mm) in experimental groups

Experimental Groups (n=20)	Mean (SD)
A-17% EDTA	2.072 (0.9971)
B-7% citric acid	1.252 (0.7205)
C-20% citric acid	1.072 (0.8852)

The coronal 3 mm of the canals in all groups were sealed with Cavisol (Golchai, Tehran, Iran) temporary filling. To ensure setting of the sealer in the experimental groups, the samples were kept in 100% humidity with 37ºC temperature for 48 hours. Then all surfaces of the teeth except for the 2-3 mm of apical root were sealed using two coats of nail polish and one coat of sticky wax (experimental and positive control groups). In negative control group, all surfaces of the teeth including the 2-3 mm of apical root were completely sealed with nail polish and sticky wax.

The teeth in the three experimental groups and two control groups were placed in 2% methylene blue dye and kept in incubator for 72 hours. After removing the samples from the incubator, they were thoroughly washed with water and the nail polish and sticky wax were removed from the surfaces. A diamond disk was used to make buccal and lingual grooves on the root surfaces; using a spatula the roots were separated into two parts and the gutta-percha and filling materials were removed from the canals. The linear dye penetration (maximum point) was measured in one-thousandth of millimeters using a stereomicroscope (MBC-10, St. Petersburg, Russia) and with the help of a computer software (Motic Images Plus 2.0 ML, Motic Instrument Inc., Canada). The data collected were statistically analyzed by One-way Variance and Post Hoc Tukey tests.

## Results

In the positive control group, the dye leaked throughout the canal in all samples. In the negative control group, penetration of dye was not observed in any of the samples. The maximum mean dye penetration was in group A with 2.072 mm and the minimum mean dye penetration was for group C with 1.072 mm (Table 1). The difference in mean linear dye leakage between groups "A and B" and "A and C" was statistically significant (P=0.012 and P=0.002 respectively), but not significant between groups "B and C" (P=0.792).

## Discussion

A three dimensional obturation and complete coronal and apical seal is one of the important aims of root canal treatment. Since microorganisms may remain in the root canal system after instrumentation, a tight apical seal is desired to prevent bacteria and their by- products from invading the apex. A perfect apical seal is also desired to prevent apical percolation.

In this regard, smear layer is one of the factors that can affect coronal and apical microleakage and thus compromise the long term success of endodontic treatment.

Since the smear layer prevents the complete locking and adherence of the root canal filling materials to the dentinal wall, many studies recommend the removal of this layer before the obturation phase of the treatment (5,9-15). Farhad and Elahi showed that Roth's 801 sealer had a significantly better apical seal when the smear layer was removed (13). Economides *et al. *indicated that resin-based sealers have superior apical sealing ability with the removal of smear layer (14). In a coronal bacterial leakage study, Farhad *et al. *demonstrated that AH26, Roth's 801, and pure ZOE sealer have better coronal seal against bacterial penetration when the smear layer was removed, and the best results were obtained when AH26 sealer was coupled with the removal of smear layer (12). The advantage of resin-based sealers like AH26 over ZOE-based sealers is that they can not only lock into open dentinal tubules but also adhere to the exposed dentinal surfaces. This characteristic of resin-based sealers is similar to the adherence capacity of composites to the dentin and enamel of teeth.

Different irrigation protocols have been introduced to remove the smear layer during root canal treatment. These irrigation regimens can create dentinal surfaces which are very different structurally. The ideal purpose is to create a particular surface of dentin which is more suitable for the specific sealer used in the obturation of the root canal system. The hypothesis is that if a dentinal surface and a sealer can complete and complement each other characteristically, ultimately they can produce a better hermetic coronal and apical seal. Considering the qualities of AH26, if the surface area of dentin exposed to this sealer is increased, the adhering and penetrating capacity of AH26 is improved and better seal is expected. In this regard, irrigation solutions which cause more erosion of dentinal wall and create a porous etched surface would be a reasonable choice. Khademi *et al. *demonstrated that 20% citric acid produces more erosion of dentin compared to 7% citric acid and 17% EDTA (16). Reis *et al. *showed that the chelating effect of 1, 5, and 10% citric acid is significantly more than 17% EDTA (18). Machado-Silveiro *et al. *in their study indicated that the decalcifying effect of 10% citric acid on dentin is more than 17% EDTA (19). Dilenarda *et al. *concluded that 1mol/L citric acid is more effective than 15% EDTA in removing the smear layer (20). On the other hand, Khedmat and Shokohinejad showed that there was no statistical difference between 7% citric acid and 17% EDTA for removal of smear layer (21). Also, Yamada *et al. *indicated that 17% EDTA is more effective in removing the smear layer than 25% citric acid (22). The factors that may be considered in obtaining these conflicting results are: type of teeth used, the concentration of the irrigation solutions, the order the irrigation solutions were used, time span of irrigation, and the criteria considered by the investigators as being effective and successful. This study was designed to test the apical sealing ability of AH26 when three different irrigation protocols were used to remove the smear layer. The results of this investigation showed that AH26 sealer has significantly better apical seal when 20% or 7% citric acid was used to remove the smear layer compared to 17% EDTA. The reason for this difference is that 17% EDTA opens the dentinal tubules with minimal changes to surrounding dentin structure, but 20% and 7% citric acid cause more erosion of the dentinal wall creating more adhesion surface area for a resin-based sealer (Figure 1). The difference between 20% citric acid group and 7% citric acid group was not significant, but AH26 showed the best resistance against apical dye penetration when it was coupled with 20% citric acid irrigation regimen. The improvement in 20% citric acid group compared to 7% citric acid group could be attributed to the fact that 20% citric acid causes more etching of the dentin and thus creating a more favorable surface for AH26 to achieve a better apical seal.

The results obtained in this study are in complete agreement with our previous investigation. In our study (17), the coronal sealing ability of two sealers (AH26 and TubliSeal) against microleakage of *Entrococcus faecalis *was compared using the same irrigation protocols mentioned above for removing the smear layer; it was shown that:

a) 20% and 7% citric acid groups in association with AH26 had the best resistance against coronal bacterial leakage,

b) 20% citric acid coupled with AH26 had the least coronal microleakage, and

c) 20% citric acid in association with TubliSeal had the greatest coronal microleakage.

Different methods such as electrochemical, radioisotope spectrometry, radiolabeled isotopes, bacterial leakage, and dye leakage techniques have been introduced to evaluate the apical seal. Because of its simplicity and low cost, dye leakage studies are popular tests. If the unwanted variables are eliminated and the experimental conditions are standardized, dye leakage studies can prove valid (23). Since dye molecules are much smaller than bacteria, investigations using dye penetration method may be less applicable to *in vivo *conditions compared to bacterial leakage techniques.

## CONCLUSION

In summary, the results of this *in vitro *study indicate that when a resin-based sealer is used for obturation of the root canal system, it may be a reasonable and smart choice to use citric acid to remove the smear layer. Furthermore, it can be concluded that the main purpose of removing the smear layer is not only to clean the dentinal tubules from organic and inorganic debris, but also to create a dentinal surface structure that best fits the characteristics of the sealer used.
